# Foreign Body Extraction Device

**DOI:** 10.1155/DTE.5.253

**Published:** 1999

**Authors:** A. Lavy

**Affiliations:** Gastroenterology Unit Rambam Medical Center Haifa Israel

## Abstract

Foreign bodies in the gastrointestinal tract are common. Mostly they are swallowed accidentally by children but also by adults. When round and slippery, foreign bodies cause a technical problem for removal. After several days in the stomach they are covered with mucus and extraction becomes even harder. There are various devices designed for use through the flexible endoscope for grasping foreign bodies but due to the great variety of objects, one may face a real problem while trying to remove them. We faced a challenge in a woman who underwent vertical band gastroplasty and was obstructed by a round and slippery hazelnut. We managed to remove the nut using a simple homemade device. This device is easy to make, cheap, and simple to use and maybe useful for various foreign bodies.


*Diagnostic and Therapeutic Endoscopy*, Vol. 5, pp. 253–255
Reprints available directly from the publisher
Photocopying permitted by license only

© 1999 OPA (Overseas Publishers Association) N.V.
Published by license under
the Harwood Academic Publishers imprint,
part of The Gordon and Breach Publishing Group.
Printed in Malaysia.

### Foreign Body Extraction Device

A. LAVY

*Gastroenterology Unit, Rambam Medical Center, Haifa, Israel*

*(Received 17 November 1998; Revised 28 January 1999; In final form 22 February 1999)*

**Foreign bodies in the gastrointestinal tract are common. Mostly they are swallowed accidentally by children but also by adults. When round and slippery, foreign bodies cause a technical problem for removal. After several days in the stomach they are covered with mucus and extraction becomes even harder. There are various devices designed for use through the flexible endoscope for grasping foreign bodies but due to the great variety of objects, one may face a real problem while trying to remove them. We faced a challenge in a woman who underwent vertical band gastroplasty and was obstructed by a round and slippery hazelnut. We managed to remove the nut using a simple homemade device. This device is easy to make, cheap, and simple to use and maybe useful for various foreign bodies.**

*Keywords:* Endoscopy, Extraction device, Foreign body

### INTRODUCTION

Foreign bodies in the gastrointestinal tract, are a common problem [1,2]. Rarely, they may cause obstruction either of the pylorus, or terminal ileum. Various devices were designed in order to deal with this problem [3–5] but sometimes for unusual objects one needs creativity to be able to remove them.

### CASE REPORT

A fifty year old woman presented with gastric outlet obstruction. She was vomiting for several days and was unable to swallow anything. Six years previously she underwent vertical band gastroplasty for morbid obesity and was doing well. Upon admission gastrograffin swallow revealed almost complete obstruction of the ring.

Gastroscopy revealed a hazelnut stuck in the ring. The nut was round and slippery and our efforts to grasp it, using various grasping forceps and a basket, were unsuccessful. Therefore we designed a suction device from a regular baby bottle nipple, put it on top of the endoscope (Fig. 1). There was no problem to inset the endoscope, as the device is of soft flexible material . We then removed the nut easily applying suction.

### DISCUSSION

Foreign bodies in the gastrointestinal tract are a common problem causing morbidity and even

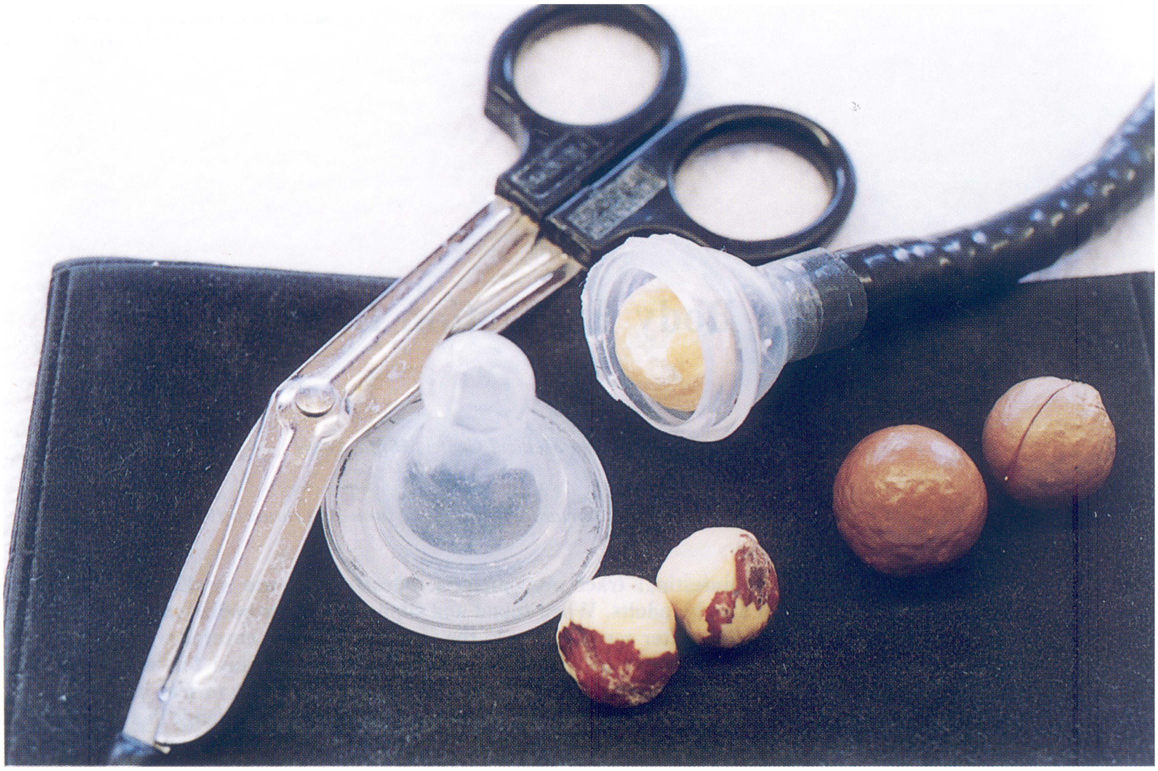

FIGURE 1 Extraction device.

mortality [6–9]. It is estimated that 1000–2000 people die in the U.S.A. from related complications [10].

Though some foreign bodies may pass through the alimentary canal without causing any harm, others depending on their size and patient's anatomy, may cause obstruction. The most common sites for obstruction are the pylorus and the terminal ileum, but in patients who underwent surgery this may vary.

Foreign bodies cause perforations, local ulcers and pain. Because of their variety, extraction should be tailored individually and indeed there are various kinds of grasping devices [11–14].

Even so, successful extraction, without damage to the esophagus needs endoscopic skill, patience and creativity.

We suggest a new home made simple device useful for some stubborn cases.

### *References*

[1] Carp, L. Foreign bodies in the gastrointestinal tracts of psychotic patients. *Arch. Surg.* 1950; **60**: 1055–1075.

[2] McCaffery, T.D. and Lilly, J.O. The management of foreign affairs of the GI tract. *Am. J. Dig. Dis.* 1975; **20**: 121–126.

[3] Rodgers, B.H.G. A new method for extraction of impacted meat from the esophagus utilizing a flexible fiberoptic panendoscope and an overtube. *Gasrtointes. Endos.* 1979; **25**: 47–48.

[4] Christie, D.L. and Ament, M.E. Removal of foreign bodies from esophagus and stomach with flexible fiberoptic panendoscopes. *Pediatrics* 1976; **57**: 931–934.

[5] Waye, J.D. Removal of foreign bodies from the upper intestinal tract with fiberoptic instruments. *Am. J. Gastroenterol.* 1976; **65**: 557–559.

[6] Maleki, M. and Evans, W.E. Foreign body perforation of the intestinal tract. *Arch. Surg.* 1970; **101**: 475–457.

[7] McManus, J.E. Perforation of the intestine by ingested foreign bodies. *Am. J. Surg.* 1941; **53**: 393–396.

[8] Edridge, W.W. Foreign bodies in the gastrointestinal tract. *JAMA* 1961; **178**: 665–668.

[9] Dagradi, A.E. and Severance, S.R. Fiberoptic endoscopic extraction of foreign body perforating the stomach. *Am. J. Gastroenterol.* 1976; **65**: 335–338.

[10] Hamillton, J.K. and Polter, D.E. Gastrointestinal foreign bodies. In: *Sleisenger & Fordtran's Gastrointestinal Dis.* W.B. Saunders: Philadelphia, Pennsylvania, U.S.A., 1995: pp. 286–292.

[11] Karaca, I. and Toller, M. Swallowed hypodermic needle in the stomach. *Oral Surg. Oral Med. Oral Pathol.* 1993; **75**: 661.

[12] Bertoni, G., Pacchione, D., Sassatelli, R. *et al.* A new protector device for safe endoscopic removal of sharp gastroesophageal foreign bodies in infants. *J. Pediatr. Gastroenterol. Nutr.* 1993; **16**: 393–396.

[13] Haug, R.H., Kimberly, D. and Brandt, C.P. Management of an ingested iatrogenic foreign body: report of a case. *J. Oral Maxillofac. Surg.* 1993; **5**: 593–596.

[14] Paul, R.I., Christoffel, K.K., Binns, H.J. *et al.* Foreign body ingestions in children: risk of complication varies with site of initial health care contact. *Pediatrics* 1993; **91**: 121–127.

